# The interactome of the amyloid β precursor protein family members is shaped by phosphorylation of their intracellular domains

**DOI:** 10.1186/1750-1326-4-28

**Published:** 2009-07-14

**Authors:** Robert Tamayev, Dawang Zhou, Luciano D'Adamio

**Affiliations:** 1 Department of Microbiology and Immunology, Albert Einstein College of Medicine, 1300 Morris Park Ave, Bronx, NY 10461, USA

## Abstract

**Background:**

Brain tissue from patients with Alzheimer's disease has shown an increase of phosphorylation of Tyr-682, located on the conserved Y682ENPTY motif, and Thr-668 residues, both in the intracellular domain (AID) of amyloid β precursor protein (APP), although the role of these two residues is not yet known.

**Results:**

Here, we report that the phosphorylation status of Tyr-682, and in some cases Thr-668, shapes the APP interactome. It creates a docking site for SH2-domain containing proteins, such as ShcA, ShcB, ShcC, Grb7, Grb2, as well as adapter proteins, such as Crk and Nck, that regulate important biological processes, cytosolic tyrosine kinases, such as Abl, Lyn and Src, which regulate signal transduction pathways, and enzymes that control phosphatidylinositols levels and signaling, such as PLC-γ. At the same time, it either reduces (like for JIP1, NUMB, NUMBL and ARH) or abolishes (like for Fe65, Fe65L1 and Fe65L2) binding of other APP interactors. Phosphorylation of Thr-668, unlike Tyr-682, does not seem to affect APP's ability to interact with the various proteins, with Pin1 and X11 being the exclusions. We also found that there are some differences between the interactions to AID and to ALID1 and ALID2, its two homologues.

**Conclusion:**

Our data indicates that APP can regulate diverse cellular processes and that, vice versa, a network of signaling events can impact APP processing. Our results also suggest that phosphorylation of the APP Intracellular Domain will dramatically shape the APP interactome and, consequently, will regulate APP processing, APP transport and APP/AID-mediated functions.

## Background

APP is a ubiquitous, type I transmembrane protein that undergoes a series of proteolytic events [1,2]. APP is cleaved by β-secretase, [3] releasing the ectodomain (sAPPβ), while the COOH-terminal fragment of 99 amino acids (C99) remains membrane bound. This is followed by an intramembranous proteolytic event, where C99 is cleaved by the γ-secretase to produce Aβ peptides plus the APP intracellular Domain (AID/AICD). Alternatively, α-secretase cleaves APP in the Aβ sequence into sAPPα and the membrane bound COOH-terminal fragment of 83 amino acids (C83), which is also cleaved by γ-secretase into P3 (the COOH-terminal Aβ segment) and AID.

The intracellular region of APP is a target for caspases, which cut APP between Asp-664 and Ser-665 [4-6] (all numbering is according to the APP695 neuronal isoform) releasing the COOH-terminal 31 amino acids of APP (C31) and the membrane bound APPΔC31. The APP intracellular peptides derived by either γ-secretase or caspase processing have biological functions. AID can modulate cell death [7-11], gene transcription [8,12-20] and Ca++ homeostasis [21,22].

The cytosolic region of APP is relatively short and does not contain motifs that may hint to an enzymatic function. Therefore, it is reasonable to postulate that cytosolic proteins that bind to the intracellular tail of APP may modulate APP/AID functions. These interactors may regulate APP trafficking and sorting and, in turn, processing. Interactions with adapters of signal transduction may link APP to intracellular signaling pathways. It is also possible that the AID released by γ-cleavage forms functional complexes with bound molecules. The following *in vitro *studies support these models: X11 and Fe65 family of proteins modulate cellular trafficking and processing of APP [23,24]. JIP1, a c-Jun N-terminal kinase JNK-signaling scaffold that binds APP [25,26], facilitates phosphorylation of APP on Thr-668 [27], functions as an adaptor protein in APP axonal trafficking [28-30] and cooperates with AID in mediating transcription [19]. The peptidyl-prolyl *cis/trans *isomerase Pin1, which binds APP only when phosphorylated on Thr668, regulates APP processing and Pin1^-/- ^mice show Tau and Aβ-related pathologies [31,32]. The putative transcriptional role of AID has attracted most of the attention because of the functional parallel with Notch signaling, another γ-secretase substrate. In the case of Notch, γ-processing releases NICD that, in the nucleus, binds transcription factors and activates transcription of specific gene targets [33,34]. For APP, a similar model has been suggested, where AID travels to the nucleus bound to Fe65, an APP-binding protein [35], Tip60, an histone acetyltransferase, and SET, a nucleosome assembly factor to activate transcription of target genes [13,36,16-18-41].

Interestingly, and consistent with the original findings implicating AID in programmed cell death [7], primary neurons derived from AID transgenic mice show increased sensitivity to certain apoptotic stimuli [11]. Therefore, there is intense interest in understanding cellular and molecular mechanisms of *in vivo *APP and AID functions. Data point to an important functional role for the phosphorylation sites Thr-668 and Tyr-682, which are found in the cytoplasmic tail of APP. Tyr-682 is included into a canonical endocytic signal motif (Y^682^ENPTY^687^) for membrane-associated receptors [42] and is important for interactions with cytosolic proteins that regulate APP metabolism and signaling [23]. Phosphorylation of Tyr-682 either promotes [43,44] or abolishes binding of some APP interactors [45]. Notably, Tyr-682 is phosphorylated *in vivo*[46,47] and this phosphorylation is abnormally enhanced in AD brain, suggesting a pathogenic role [48,49]. Thr-668 is followed by a Pro [50], which generates a consensus site for phosphorylation. Phosphorylation of Thr-668 generates a docking site for Pin1 and this interaction may contribute to AD pathogenesis [31,32,51]. Conversely, Thr-668 phosphorylation reduces binding of Fe65 to APP[45]. Remarkably, the phosphorylation of Thr668 is increased in AD brains [49].

APP belongs to a gene family that includes APLP1 and APLP2. These three proteins are structurally similar and share many functional similarities. They are similarly processed by secretases and caspases; the YENPTY motif is shared by all three APP family members, is evolutionally conserved, and generates docking site for common interactors; the TP phosphorylation site is conserved in APP family members and in other species, except for APLP1 and Drosophila APP ortologue. In spite of all these similarities however, APLP1 and APLP2 have not been involved in neurodegeneration. It is hypothesized that β/γ-cleavage of APLPs does not generate Aβ-like peptides, prone to oligomerization and amyloid formation, explaining why they have not been linked to AD or AD-like dementias. The evidence that AID regulates apoptosis prompted us to postulate that deregulations of AID production may participate in AD pathogenesis [7]. In this context, functions that are specific for AID and are not shared by the corresponding ALID1 and ALID2 fragments (which are generated by γ-cleavage of either APLP1 or APLP2) may be involved in neurodegeneration.

The SH2 domain proteins that we studied were ShcA, ShcB, Grb7, Grb2, Crk, Nck, p85, Abl, Lyn, Src, SHIP-2, PLCγ. Shc and Grb2 have been found to interact with APP, requiring phosphorylation of APP at Tyr-682 [20,43,44,52]. This could lead to the activation of the MAPK pathway, since Shc and Grb2 are known to link growth factor receptors to signaling pathways, such as Ras, MAPK, and PI3K, and participate in oncogenic proliferation, neuronal development, cell differentiation, and apoptosis [52-57]. Grb7, Crk, and Nck, along with Grb2, are adaptors with SH2 domains. Crk is believed to be necessary to complete cytokinesis, although the details are not well known [58]. Crk also plays a role in a complex with C3G, Rap1, and B-Raf that has EGF activating MAPKs, whose cascade links cell surface receptors to cytoplasmic and nuclear effectors [59-61]. The SH2 domain of Crk has been found to bind to a tyrosine in the EGF receptor [61]. Nck, like Crk, contains both an SH2 and SH3 domain and plays a role in regulating tyrosine kinase signaling. Nck is believed to have roles in actin cytoskeleton, cell movement, and axonal guidance [62]. In sporadic AD, human Nck associated protein 1's (Nap1) expression is downregulated, leading to apoptosis in human neuroblastoma cells. Nap1, and its binding protein hNap1BP, might have a role in regulating β-secretase activity; Human Nap1 and hNap1BP increased the level of sAPPα [63,64].

Abl is one of the three tyrosine kinases that belong to the Src family of protein kinases that we chose to test. Expression of the active form of Abl in cell lines triggers, either directly or indirectly, phosphorylation of Tyr-682 of APP, and Abl can form a stable complex with APP [47,65]. The second kinase tested is Lyn, which is part of the Src family and is a modulator of the molecular events initiated by engagement of the high-affinity IgE receptor. It is also believed that Lyn is linked to lipid kinases and have a role in lipid second messengers that control mast cell function and allergic responses [66]. Another kinase tested was Src, whose exact roles are not well known, however it is known to be a tyrosine kinase that is found in viruses and cells. Src may have a role in mitosis, intracellular localization, and has SH2 and SH3 domains that have important roles, as there is a phenotype to cells who have those domains mutated [67].

We chose to test SHIP-2, since it is a phosphoinositide phosphatase recently implicated as a negative modulator of insulin signaling [68]. SHIP-2 is a phosphatase that negatively regulates the JAK/STAT signaling pathway by downregulating JAK activity [69]. PLCγ is known to have roles in cell signaling, responding to extracellular stimuli, including hormones, neurotransmitters, antigens and growth factors. PLCγ acts to catalyze the hydrolysis of phosphatidylinositol (4,5)-bisphosphate [PtdIns (4,5)P(2)], releasing two well-known second messengers – inositol (1,4,5)-trisphosphate and diacylglycerol – which have numerous roles as well [70].

The PTB domains that we have addressed include JIP1, JIP2, AIDa, ARH, DAB1, DAB2, ShcC, ShcA, NUMB-p71, NUMB-p72, NUMB-Like, X11α, X11β and X11γ. JIP1a, JIP1b, and JIP2 are PTB domain containing scaffold proteins that interact with the -YENPTY- motif of APP and the binding is phosphorylation independent [25,26,28,71]. JIP1 and JIP2 are both able to bind parts of the JNK signaling pathway [72-74]. JIP1b was found to bind APP, and the binding is increased when Thr-668 is phosphorylated by JNK and APP is associated with kinesin light chain 1 (KLC1) [28,30]. This may show that JIP1 links KLC1 to APP [28]. DAB1-PTB domain binding to the cytoplasmic tail of APP *in vitro *and in cells has been shown with high affinity, but is inhibited by phosphorylation of APP [75]. DAB1 has also been shown to interact with APLP1, and weakly interact with APLP2 [76]. APP and APLP1 are able to increase the serine phosphorylation of DAB1, which might function through a link with APLP1 in the brain [76]. This is further proven by showing that APLP1 and DAB1 are expressed in overlapping cell populations in brain tissues [76].

The NUMB family was studied due to its effects on Notch. APP has been found to bind NUMB through its PTB domain in a phosphorylation independent manner [20]. We have previously shown that AID binds NUMB and NUMB-L, and represses Notch activity when released by APP [20]. Notch has been suggested to play a role in physiological and pathological cell death, where its overexpression protects T-cells from apoptosis [77]. Some isoforms of NUMB are able to sort APP to the recycling and degradative pathways and has roles in APP metabolism [78]. X11α, X11β and X11γ are all part of the X11 family and are adaptor proteins that are known to stabilize APP, which prevents its cleavage by β and γ-secretases [79]. X11α and β are expressed in neurons, while X11γ is expressed ubiquitously. X11 has been found to bind the YENPTY motif of AID and the binding is phosphorylation independent [80]. X11α and X11β are both able to bind to munc18, a synaptic vesicle docking protein, which is vital for Ca^++^-mediated synaptic vesicle exocytosis, showing that X11s have roles in synaptic vesicle docking and exocytosis [81].

Here, we use a direct biochemical approach to elucidate the following points: 1) characterize the AID, ALID1 and ALID 2 interactome; 2) assess the role of Tyr-682 and Thr-668 phosphorylation in shaping this interactome; 3) identify interactions that are specific to AID. Our results indicate that both Tyr-682 and Thr-668 impact the complex APP-Intracellular Domain Interactome, however, the effect of Tyr-682 phosphorylation is more dramatic. In fact, phospho-Tyr-682 becomes a docking site for proteins containing a Src-hmology2 domain (SH2) while it either reduces or obliterates interaction of a subset of proteins containing a Phospho-Tyrosine-Binding (PTB) domain. This study is an obligatory starting point to understand the biochemical mechanisms of AID functions, to elucidate the physiological role of APP phosphorylation, and to identify signaling pathways that may go awry in AD.

## Materials and methods

### Plasmids and Cloning

For APP intracellular domain (AID) expression in mammalian cell lines, an Fc-fusion construct coding for the last 50 residues of APP was generated.

### In Vitro Protein Pull-Down Assays

Equivalent molar amounts (3 nmol) of strep-tag AID peptides were incubated with 30 μl of 50% Strep-Tactin matrix (IBA) in a total volume of 400 μl of NET-N buffer (150 mM NaCl, 1 mM EDTA, 50 mM Tris/HCl, 1% (v/v) Nonidet P-40, pH 8.0) for 1 h at 4 °C. The beads were washed two times with 400 μl of NET-N buffer and then incubated with μg of each GST fusion protein in 400 μl of NET-N buffer for 2–4 h at 4°C. The beads were then washed with 1 ml of NET-N four times. The bound proteins were eluted from the beads by boiling the samples at 95 °C in SDS-PAGE loading buffer for 4 min. Proteins were analyzed by NuPAGE^® ^Novex Bis-Tris 4–12% gel (Invitrogen) electrophoresis, and then each gel was stained with Coomassie Blue.

### In-Vitro Protein Interaction with Strep-tag AID peptides, GST-Pin1, GST-Grb2, GST-Crk, and Fc-AID, Pulldowns

The last 50 amino acids of APP were synthesized either as non-phosphorylated (AID) or Thr-668-phosphorylated (AIDpT) APP peptides with an N-terminal strep-tag (Tufts University Core Facility Boston, MA), and have been described previously [43]. The strep-tagged peptides were immobilized on Strep-Tactin column (IBA, St. Louis, MO) and incubated with Strep-Tactin precleared AD brain homogenates. After washing, samples were eluted with 10 mM desthiobiotin as per manufacturer's recommendation [82]. For GST-pull-downs, recombinant GST-Pin1, GST-Grb2, GST-Crk immobilized on Glutathione Sepharose were incubated with Strep- Tactin bound strep-tagged AID peptides, and processed as described [43]. The bound proteins were washed, eluted by boiling in sample buffer, and analyzed by Coommassie Blue staining. N2a cells were transiently transfected with Fc-AID for 48 hours, and prior to harvest were treated in the presence or absence of 10 mg/mL anisomycin (Sigma) for 30 min. Lysates were incubated with Protein G Sepharose 4Fast Flow™ beads Amersham), and following washes, bound proteins were eluted in hot sample buffer. Precipitates were analyzed by immunoblotting or Coommassie Blue staining. ImageJ was used to quantitated the percentage of binding.

### BIAcore Assays (surface plasmon resonance biosensor assay)

Binding of GST and GST-Grb2, NUMB p71, and X11β domains to strep-tag AID peptide or different phosphorylation forms was monitored by surface plasmon resonance (SPR) on a BIAcore 3000 machine (BIAcore, Neuchâtel, Switzerland). The strep-tag peptide and different phosphorylation forms of AID peptides were covalently amine coupled to a CM-5 sensor chip by use of the amine covalent coupling. An immobilization level of 4500–7500 resonance units was obtained. A nonderivatized flowcell serves as a reference surface. Interactions between GST and GST-Grb2, NUMB p71, and X11β to strep-tag peptides were determined by the change in signal measured in RU. Between each sample examined, the surfaces were regenerated with a 1-minute pulse of 50-mM glycine-NaOH buffer (pH 8.5) that resulted in complete dissociation of non-covalently bound analyte (GST and GST-Grb2, NUMB p71, and X11β).

## Results

### Phosphorylation of APP governs binding to proteins containing SH2 domains

The intracellular region of APP involved in binding the vast majority of known interactors includes the YENPTY motif and Thr-668. Most of the cytosolic interactors of APP bind these sequences through a PTB or an SH2 domain. One notable exception is represented by Pin1, which is not known to bind through a PTB or an SH2 [32]. To directly test how phosphorylation of Tyr-682 and Thr-668 regulate the intracellular interactome of APP, we have synthesized the AID peptide as well as phosphorylated AID peptides on Thr-668 (AIDpT), Tyr-682 (AIDpY), and both (AIDpTpY). These AIDpeptides were fused to the strep-tag sequence. A control peptide, consisting of the streptag sequence only, was also made. We produced a series of known or potential APPbinding proteins *in vitro*, using just the regions with the SH2 and/or PTB domains. All these targets analyzed contain either an SH2 or a PTB domain fused to GST for production and purification from bacterial cultures. The AID peptides were immobilized on Strep-Tactin resin and challenged with 6 μg of recombinant GST-fusion proteins. The control was GST on its own, and it did not bind any protein (data not shown).

**Figure 1:**
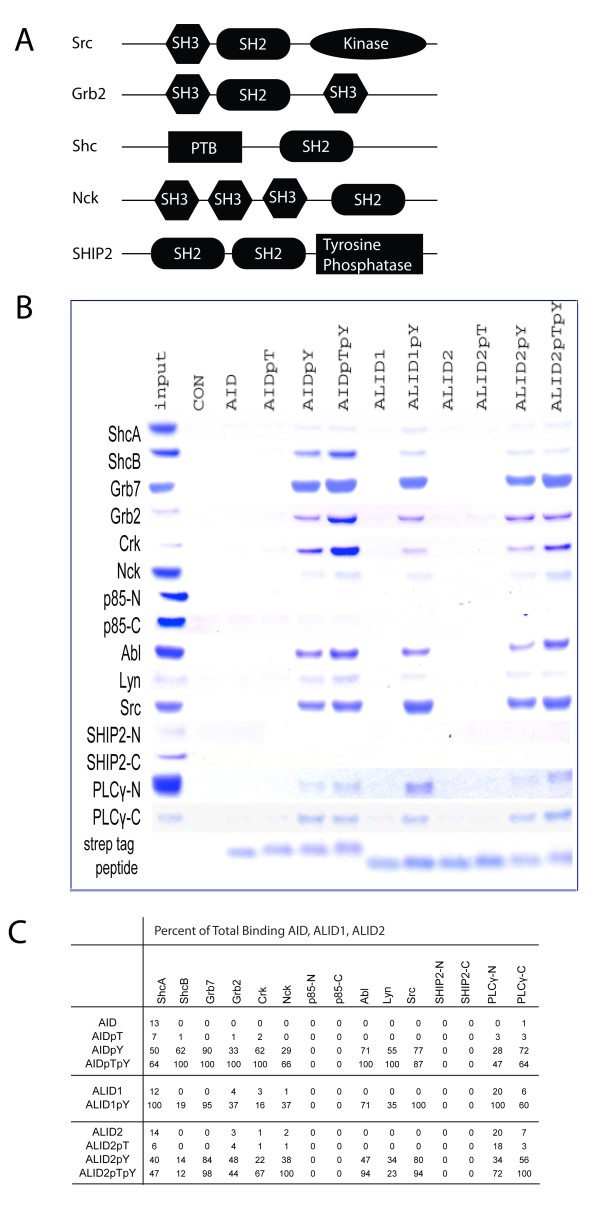
**Effect of phosphorylation of Thr-668 and Tyr-682 on APP, APLP1, and APLP2 to SH2 domain containing proteins**. (A) Schematic diagrams of the structural domains of the various SH2 domain peptides studied. (B) APP C-terminal (AID), APLP1 C-terminal (ALID1), and APLP2 C-terminal (ALID2) and strep-tag control peptides were synthesized. Pull down experiments with immobilized Strep-Tactin resin tested the binding of AID, ALID1, ALID2, and their phosphorylated counterparts to ShcA, ShcB, Grb7, Grb2, Crk, Nck, p85-N, p85-C, Abl, Lyn, Src, SHIP2-N, SHIP2-C, PLCγ-N, and PLCγ-C. The pull-down samples were analyzed by SDS-PAGE and Comassie-blue staining. (C) Percent binding of the AID, ALID1, ALID2 and the phosphorylated ones to the SH2 domain-containing proteins listed above. The interaction that has the greatest binding affinity was designated 100% and the others were represented as a fraction of the maximum binding. All percentages were rounded to the nearest whole number.

We show that the adaptor proteins, including ShcA, ShcB, ShcC, Grb2, Grb7, Crk, and Nck, all bind in a similar fashion. They bind to AID when Tyr-682 is phosphorylated, and that binding is further increased when Thr-668 is also phosphorylated (Figure 1b, c). When testing p-85, a regulatory subunit for class I phosphoinositide 3-kinase (PI3K), both the N-terminus and C-terminal SH2 domains of p-85 showed no binding to AID, whether Thr-668 and/or Tyr-682 were phosphorylated (Figure 1b).

The tyrosine kinases tested, Abl, Lyn, and Src, followed the same trend as the adaptors, being unable to bind to unphosphorylated AID or AID when only Thr-668 is phosphorylated, showed binding when Tyr-682 is phosphorylated. This binding is more intense when both residues are phosphorylated (Figure 1b, c). When testing the phosphatase SHIP-2, both its N-terminus and C-terminus bound neither unphosphorylated AID nor any of its phosphorylated counterparts (figure 1b, c). PLCγ-N and PLCγ-C were able to bind AID with the tyrosine phosphorylated and could bind with higher affinity if both the tyrosine and threonine were phosphorylated, following the trend of the adaptors and kinases (Figure 1b). In summary, the SH2 domain proteins that showed binding were dependent on the phosphorylation of tyrosine and double phosphorylation increased binding.

### Phosphorylation of APP governs binding to proteins containing PTB domains

**Figure 2:**
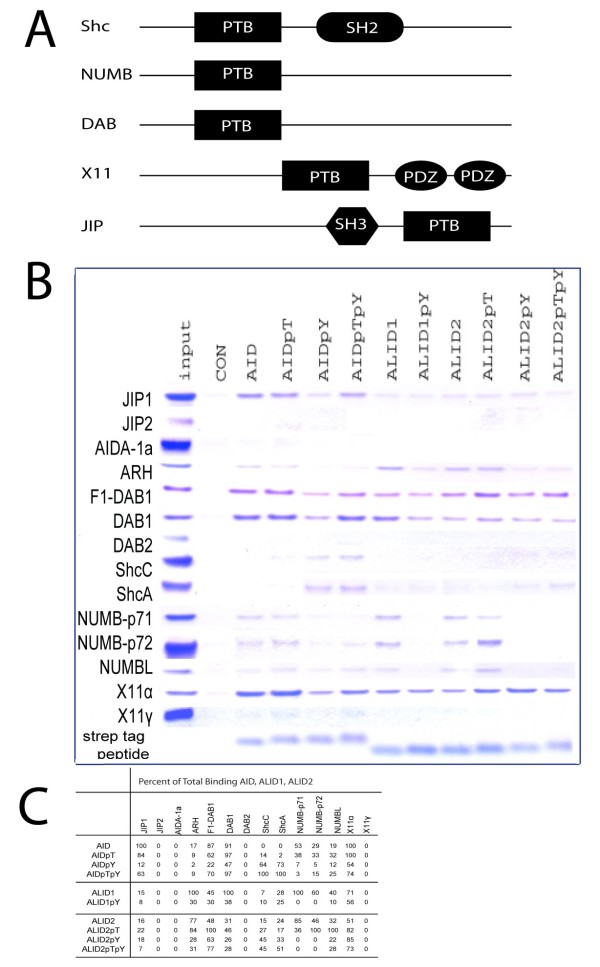
**Effect of phosphorylation of Threonine668 and Tyrosine682 on APP, APLP1, and APLP2 to PTB domain containing proteins**. (A) Schematic diagrams of the peptides containing PTB domains. (B) The same peptides as in Figure 1 were synthesized. Pull down experiments with immobilized Strep-Tactin resin showed the binding of the peptides to JIP1, JIP2, AIDA-1a, ARH, DAB1, DAB2, ShcC, ShcA, NUMB-p71, NUMB-p72, NUMB-L, X11α, and X11γ. The pull-down samples were analyzed by SDS-PAGE and Comassie-blue staining. (C) Percent binding of the AID, ALID1, ALID2 and the phosphorylated ones to the PTB domain-containing proteins listed above. The interaction that has the greatest binding affinity was designated 100% and the others were represented as a fraction of the maximum binding. All percentages were rounded to the nearest whole number.

The same peptides and strategy described above were used to analyze proteins with PTB domains, including JIP1, JIP2, AIDa, ARH, DAB1, DAB2, ShcC, ShcA, NUMBp71, NUMB-p72, NUMB-L, X11α, and X11γ. The structural domains of some of these proteins are seen in Figure 2a. JIP1-PTB, ARH, NUMB, and X11α bound unphosphorylated AID strongly. They were still able to bind AID when the threonine was phosphorylated (AID^p^T), though the binding was slightly weaker. However, the interaction was visibly reduced when either both threonine and tyrosine (AID^p^T^p^Y) or tyrosine (AID^p^Y) (figure 2b, c). DAB1-PTB and F1-DAB1 both showed that binding to AID was not decrease much when Thr-668 was phosphorylated, but as with the other PTB-containing proteins, there is a sharp decrease with the phosphorylated of Tyr-682 on its own (figure 2b, c). JIP2, AIDA-1a, DAB2, and X11γ, however, were unable to bind AID regardless of whether the threonine or the tyrosine is phosphorylated (figure 2b).

### Phosphorylation of APLP1 and APLP2 do not always show the same effects as the phosphorylation of APP

To extend our observations to the other members of the APP gene family, we synthesized corresponding APLP1 and APLP2 peptides (ALID1, ALID1^p^Y, ALID2, ALID2^p^T, ALID2^p^Y and ALID2^p^T^p^Y). ALID1^p^T and ALID1^p^T^p^Y were not made because this threonine of APLP1 is not phosphorylated [27]. The peptides were immobilized on Strep-Tactin resin and challenged with 6 μg of recombinant GST-fusion proteins. The control was GST on its own, and it did not bind any protein (data not shown).

From the SH2-containing proteins discussed above, ShcB, Crk, Grb2, and Lyn have a higher affinity for AID^p^Y than ALID1^p^Y and ALID2^p^Y. Grb7, c-Abl, Nck, PLCγ-C, and Src show no difference between AID^p^Y than ALID1^p^Y and ALID2^p^Y, while ShcA and PLCγ-N have a higher affinity for ALID1^p^Y (figure 1b, c). Like for AID, the SH2- domain containing proteins only bind ALID1 and ALID2 when the Tyr residue is phosphorylated. The binding to ALID2 is slightly increased when in addition to the tyrosine being phosphorylated, the threonine is also phosphorylated (figure 1b).

The PTB-containing domains show more variation in preference between the three homologues. JIP1 binds to AID much stronger than ALID1 and ALID2. ARH binds to ALID1 more than AID and ALID2. DAB1 binds to AID and ALID1 more than ALID2. The three forms of NUMB show preference for the ALIDs over AID. ARH, NUMB-p71, NUMB-p72 and NUMBL have a decrease in binding when tyrosine is phosphorylated. X11α shows to be phosphorylation independent. The PTB domains of ShcA and ShcC bind in a similar fashion as the SH2 domains, with a higher binding to phosphorylated AID than ALID1 and ALID2. JIP2, AIDA-1a, DAB2, and X11γ do not bind AID, ALID1, ALID2, or any of the phosphorylated peptides (figure 2b, c).

### Quantitative assay showing the effect of phosphorylation of Tyr-682 and Thr-668 on binding affinity

**Figure 3:**
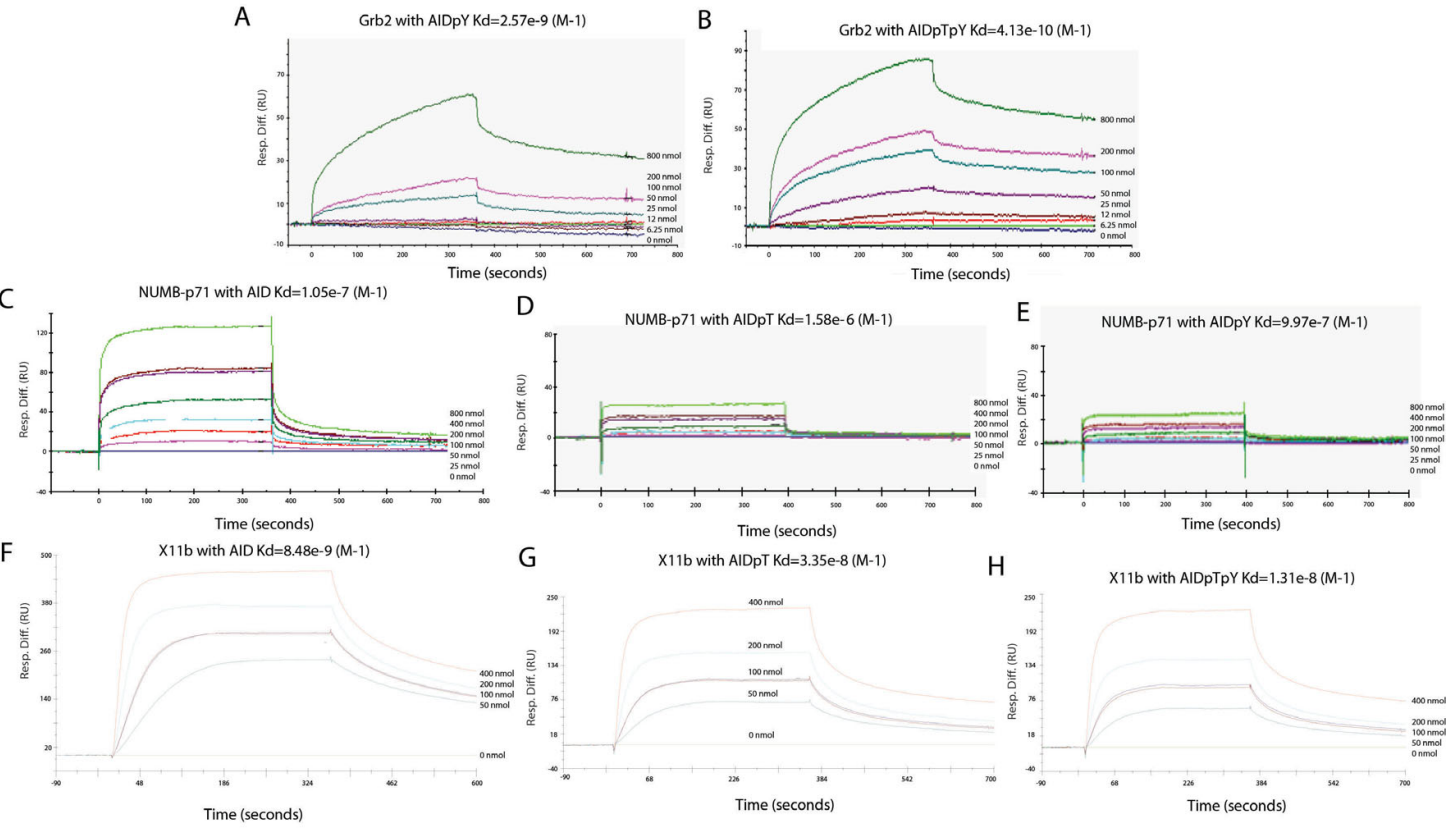
**Quantitative analysis of protein-protein interactions using Biacore assay (surface plasmon resonance biosensor assay)**. Strep-tagged AID peptides and Strep-tag control peptide were immobilized on sensor chips CM5 (Biacore). Binding curves for GST-Grb2, Numb p71, and X11β proteins were expressed in resonance units (RU) as a function of time. (A) AID^p^Y and GST-Grb2 interaction, (B) AID^p^T^p^Y and GST-Grb2 interaction, (C) AID and GSTNUMB-p71 interaction, (D) AID^p^Y and GST-NUMB-p71 interaction, (E) AID^p^T^p^Y and GST-NUMB-p71 interaction, (F) AID and X11β interaction, (G) AID^p^T and X11β interaction, and (H) AID^p^T^p^Y and X11β interaction. The kinetic parameters and concentrations of the analytes are indicated in A-H.

We utilized a quantitative method (surface plasmon resonance biosensor assay, Biacore) to precisely measure the effect of Thr-668 and Tyr-682 phosphorylation on the affinity of Grb2, NUMB-p71, and X11β for APP. These measurements allow a more accurate view of the binding affinities than the pull-down experiments conducted. We found that AID^p^Y and AID^p^T^p^Y bind to the GST-Grb2 domain with a KD of 2.57 × 10^-9 ^(M^-1^) and 4.13 × 10^-10 ^(M^-1^), respectively (Fig. 3A-B). There is not measurable binding when AID is not phosphorylated on Tyr682 (not shown). Repeating the experiment with NUMB p71, we saw that AID, AID^p^T and AID^p^Y bind to the GST-NUMB-p71 with a KD of 1.05 × 10^-7 ^(M^-1^), 1.58 × 10^-6 ^(M^-1^), and 9.97 × 10^-7 ^(M^-1^), respectively (Fig. 3C–E). And AID, AID^p^T and AID^p^T^p^Y bind to the GST-X11β with a KD of 8.48 × 10^-9 ^(M^-1^), 3.35 × 10^-8 ^(M^-1^), and 1.31 × 10^-8 ^(M^-1^), respectively (Fig. 3F–H).

These data show that Grb2 binds AID stronger when both Tyr-682 and Thr-668 are phosphorylated than when just the tyrosine phosphorylated, which supports the pull down data above. Unlike to Grb2, in the case of NUMB-p71, the binding affinity to unphosphorylated AID is strongest, decreases when tyrosine or threonine is phosphorylated independent of the other. Binding of NUMB-p71 being strongest to unphosphorylated AID is consistent with the pull down data. As for X11β, binding to AID is highest when it isn't phosphorylated, slightly lower when both tyrosine and threonine is phosphorylated and even lower when threonine alone is phosphorylated. The decrease of affinity of a domain due to the phosphorylation of the threonine has been documented by our lab before. We saw that the affinity of Fe65 to AID was decreased by a factor of 3 [45]. These data show that phosphorylated versions of the two residues can both increase and decrease the binding affinity of a domain to APP, depending on what the domain is.

### Confirming interactions with pull down experiments from brain homogenates

**Figure 4:**
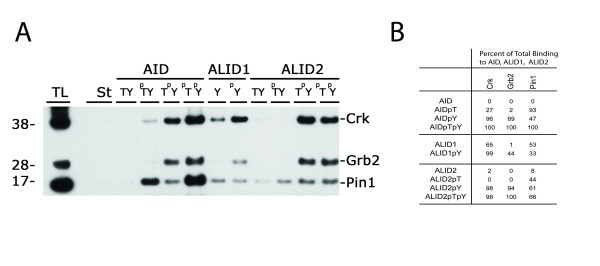
**Pull down experiment of brain homogenate**. (A) Brain homogenates were prepared and pull-down experiments were done and studied by Western Blot. The results for Grb2 and Crk confirmed those found through *in-vitro *pull down. Pin1 results show that binding is increased through the phosphorylation of Thr-668. (B) Percent binding of the AID, ALID1, ALID2 and the phosphorylated ones to the brain homogenates protein: Grb2, Crk, Pin1. The interaction that has the greatest binding affinity was designated 100% and the others were represented as a fraction of the maximum binding. All percentages were rounded to the closest whole number.

The interactions of Grb2 and Crk with phospho-tyrosine of Strep-tag AID peptide were confirmed by pull-down experiments from human brain lysates (figure 4). The results confirmed those with the GST-SH2 recombinant protein, except that Crk and Grb2 were able to bind to AID with just threonine phosphorylated and unphosphorylated ALID1 although the binding affinity was low.

Pin1 was shown to bind to phosphorylated Thr-668, which is important since knockout of Pin1 causes tauopathy and neurodegeneration and Pin1 is downregulated in AD neurons [32,83]. Pin1 knockout increases Aβ42 production, increasing amyloidogenic APP processing, showing that Pin1 might lead to non-amyloidogenic APP processing and reduce Aβ production [32]. Overexpression of Pin1 in cells causes a decrease in the amyloidogenic processing of APP [84]. Using brain homogenate, we confirmed that Pin1 is able to bind AID when only Thr-668 is phosphorylated. However, and surprisingly perhaps, Pin1 also bound to AID^p^Y and double phosphorylation on both Tyr-682 and Thr-668 augmented the amount of Pin1 interacting with APP (figure 4). As for ALID1 and ALID2, the data are not conclusive. We found some binding of Pin1 that is not affected by phosphorylation. Thus, it appears that Pin1 preferentially binds phosphorylated forms of APP (figure 4).

## Discussion

**Figure 5:**
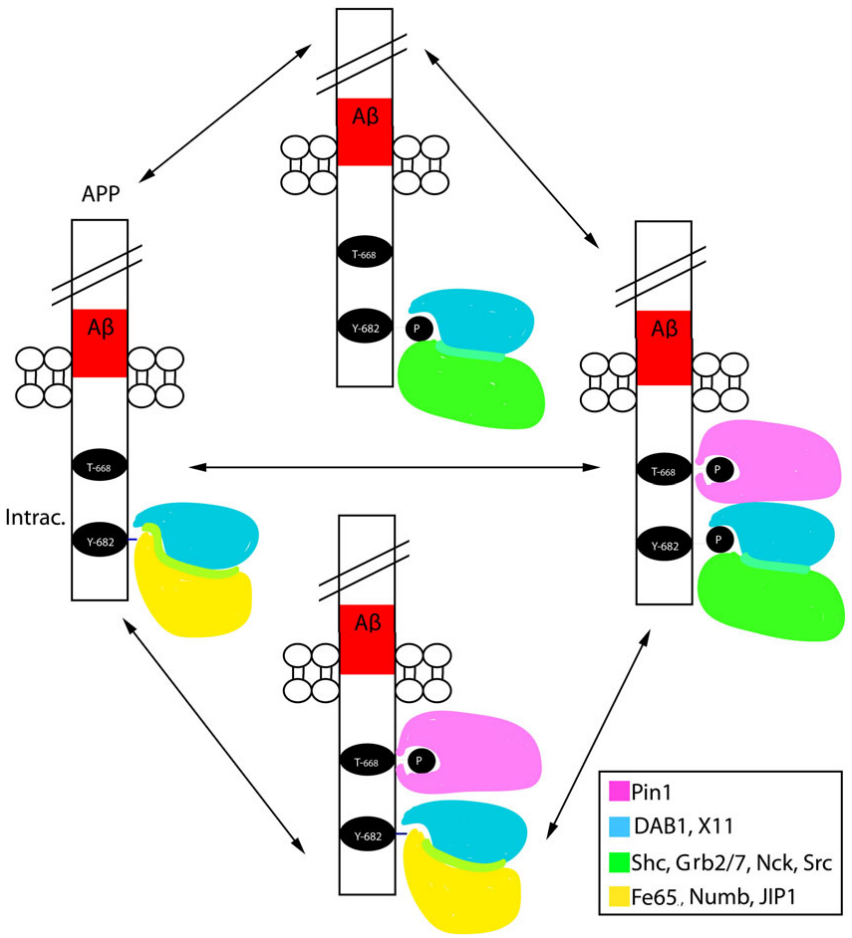
**Schematic showing the effect of phosphorylation of Tyr-682 and Thr-668 on the APP's interactome**.

In this study we show that phosphorylation of Tyr-682 and Thr-668 dramatically changes the APP interactome (Figure 5). These phosphorylations affect the ability of proteins with Src-Homology 2 domains (SH2) and Phospho-tyrosine Binding domains (PTB) bind to APP, APLP1, and APLP2. Interactions between proteins, such as the generation of docking sites, are affected by phosphorylation, so we studied various interactions that are enhanced/reduced when these two residues are phosphorylated [85]. This makes the interactions important to study since phosphorylation of these two residues is enhanced in AD brains, suggesting a pathogenic role [48,49,86]. Tyr-682 is included into a canonical endocytic signal motif (Y682ENPTY687) for membraneassociated receptors [42]. This motif is shared by all three APP family members (APP, APLP1 and APLP2), is evolutionally conserved, and is important for interactions with cytosolic proteins that regulate APP metabolism and signaling [87]. Here we show that phosphorylation of Tyr-682 dramatically changes the APP-Intracellular Domain Interactome. In fact, it creates a docking site for proteins containing an SH2 domain and either reduces or obliterates interaction of a subset of proteins containing a PTB domain.

The proteins with the SH2 domains tested were ShcA, ShcB, Grb7, Grb2, Crk, Nck, p85-N, p85-C, Abl, Lyn, Src, SHIP2-N, SHIP2-C, PLCγ-N, and PLCγ-C. Among these domains are scaffolds, adaptors, kinases, phosphatases and phospholipids second messengers. The structural domains of the listed proteins are found in Figure 1a. The proteins with the PTB domains tested were JIP1, JIP2, AIDa, ARH, DAB1, DAB2, ShcC, ShcA, NUMB-p71, NUMB-p72, NUMB-Like, X11α, X11β and X11γ. These proteins, as the SH2 domain-containing proteins, have various roles among them. Some of their structural domains are seen in Figure 2a.

**Figure 6:**
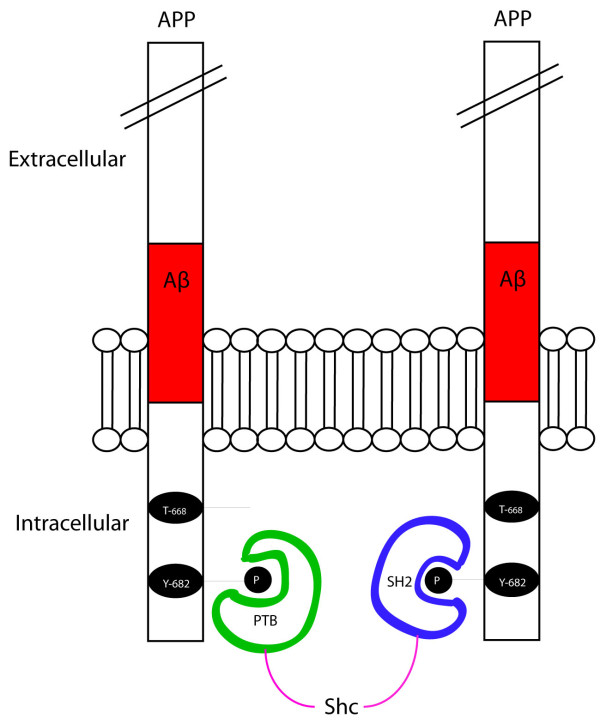
**Model of how Shc could bind to APP using both its SH2 and PTB domain, making a dimeric APP complex induced by Tyr-682 phosphorylation**.

ShcA, ShcB, and ShcC are SH2 and PTB-containing adapter proteins that signal to cellular differentiation and survival pathways. Our previous studies have shown that APP and ShcC were physically associated in adult mouse brain homogenates. We have also seen interactions of APP and the three *in vitro*, finding that they bind through the PTB domain [44]. It is believed that phosphorylation of APP might connect Shc and Grb2 to various cellular pathways, which are important to study to understand the role of APP. From the Shc proteins, ShcA is found to be higher in AD brains compared to normal brains [52]. ShcA and ShcC bind APP through their PTB domain and are different from the others PTB-domain containing proteins we studied in that they only associate with APP when Tyr-682 is phosphorylated [44,53,85,88]. ShcA, along with ShcB, also bind APP through their SH2 domain (the SH2 domain of ShcC and the PTB domain of ShcB have not been analyzed in this study). This finding may be of importance as it is possible that Shc proteins may mediate formation of dimeric complex between two APP proteins that is induced by Tyr-682 phosphorylation. A potential model is shown in Figure 6. Another interesting observation was that the SH2 domain of ShcA binds stronger to ALID1 while its PTB domain binds stronger to AID (figures 1b, c and 2b, c). Thus, it is also possible that Shc proteins could generate hetero-dimers between APP family members upon Tyr phosphorylation.

The adaptors, Grb7, Grb2, Crk, and Nck, are all seen to be dependent on phosphorylation of Tyr-682. Grb2 and Crk, unlike Grb7 and Nck, bound preferentially to AID rather than ALID1 or ALID2. This is of relevance since AD patients have the two residues phosphorylated, and APP is the one believed to play a role in AD, not ALID1 or ALID2. This leads us to the conclusion that Grb2, Crk, and also the Shc family, but not Grb7 and Nck, play a role in AD.

p-85 is a regulator'y subunit for class I phosphoinositide 3-kinase (PI3K). PI3K has various roles in human cancer [89]. Since p-85 is a dimeric enzyme that acts as a regulator, we wanted to see if it had any role in regulating APP, and so we tested its binding. Although no binding was found, it may still have a role in regulating APP through a mutual binding partner.

Abl has been shown to bind Fe65 and induce the phosphorylation of Fe65 leading to the APP/Fe65-mediated gene transcription [65]. Abl has also been found to interact with APP through its SH2 domain [47]. Unlike other kinases, Abl has a long C-terminus and holds nuclear localization, nuclear export signals, and domains that can interact with DNA and F-actin [90,91]. Lyn has been found to be recruited into a complex between APP and β1 integrin mediated adhesion of monocytes; this recruitment came along with an increase in phosphorylation of Tyr-682 of APP, which may lead to activation of cytoplasmic serine/threonine kinases and subsequent transcriptional regulation. Binding of Lyn to APP only took place when it was phosphorylated, leading to an increase in p38 MAP kinase activity [92-95]. Src kinase, along with Abl, has been found to be involved in the phosphorylation of Tyr-682, which is seen in the brain of patients with AD as well as patients without the disease [43,47]. Src and Abl bind fairly even to the three homologues, but Lyn preferentially binds AID, so it may play a role in AD.

SHIP2 is able to activate Ras through Grb2 and SOS, leading to the phosphorylation and activation of Raf-1/MEK-1/MAP kinases [96,97]. Howell et al found that DAB1-PTB interacts with SHIP; the binding is inhibited if the tyrosine residues of the ligands are phosphorylated [75]. Just as with p-85, there is no binding between SHIP2 and the APP family but it may bind through an adaptor such as Grb2.

PI-PLC plays a general role in removing GPI-anchored proteins from the cell surface. There is some evidence that GPI-anchored proteins play a part in β-secretase activity and Aβ secretion, though no real role in α-secretase activity [98]. PI-PLC treated CHO cells have a reduction in the processing of beta-secretase [98]. The same lab has also shown that PI-PLC treated brain cultures show a reduction in the secretion of Aβ but not sAPPα. PLC is important to study too because it was seen that when PS1, PS2 or both are missing in MEFs, the activation of PLC, PKCα, and PKCγ activations were much lower after PLC was stimulated [99]. The authors also point out that PKCα and PKCγ protein levels were lower in the knockouts but PKCδ levels were much higher [99]. We tested both the N- and C-termini and found that they both bound to the three homologues and the binding was stronger to APLP1 and APLP2, which means it may also play a role, possibly a larger role, in the cleavage of the two homologues.

The JIP1 and 2 are scaffold proteins, which have been studied extensively in our lab. We have seen JIP interact with AID, and now we further tested the interaction with phosphorylated AID and its two homologues [27]. JIP1b stabilizes immature APP, preventing the release of AID and sAPP and secretion of Aβ-40 and Aβ-42 [100]. JIP2 does not bind APP as strongly as JIP1 and does not have an effect on APP processing [100]. JIP1 and APP were found to be transported together in vesicles when APP is phosphorylated at Thr-668, showing that the formation of a complex between JIP1 and APP is formed only when APP is phosphorylated [30]. JIP1 shows preference for AID but JIP2 doesn't bind either one; thus JIP1 may have a pathogenic role in AD.

AID-1a has been shown in previous studies in our lab to possibly having a role as a modulator of APP processing. It was shown to inhibit the function of γ-secretase, reducing the amount of Aβ secreted [101], but did not bind either homologue in this study. It has been seen that downregulating ARH expression using RNAi *in vitro *increases cellular APP levels. The interaction of ARH with AID has been seen prior both *in vitro *and *in vivo*, and gave ARH a possible role in APP internalization, transport, and/or processing [102]. It has also been shown to regulate cholesterol uptake and so might have a role on cholesterol metabolism in APP processing [102,103]. ARH binds much stronger to ALID1 and ALID2 than it does to AID. This makes it an important protein to study, because it may have a role in preventing oligomerization, since APLP1 and APLP2 do not form plaques after they are cleaved. DAB1 has been shown to be linked to APP through JIP1b, and DAB1 is believed to play a role in neuronal development [28,47,75,76]. DAB2 is able bind to clathrin coated pits and cytoskeletal components, so it might play roles in endocytic trafficking of lipoprotein receptors and cell adhesion/spreading [76,104,105]. DAB1 seems to be phosphorylation independent and DAB2 does not bind to any of the peptides. This means that DAB1 probably has a constant role, unless regulated by a third party, such as JIP1b.

NUMB isoforms that have the PTB domain were found to increase PC12 cells' susceptibility to death by Aβ-42, showing that NUMB may have roles in neural development and neuro-degenerative disorders [106]. Mice without a functional NUMB die in early development and have defects in cranial neural tube closure and premature neuron production, showing that NUMB has a role in cell survival, especially since it binds Notch and Notch has been found to promote cell survival [107]. Notch is cleaved by γ-secretase releasing the Notch Intracellular Domain (NICD), which translocates to the nucleus and activates the transcription of genes that regulate the generation, differentiation, and survival of neuronal cells. APP is able to bind to the NUMB family, which inhibits Notch, by interacting with it [20,108]; thus it was important to study the interaction of APP to NUMB. The three forms of NUMB studied were bound more to ALID1 and ALID2. In our studies, the Biacore data showed that NUMB binds AID with higher affinity than AID^p^T and AID^p^Y, consistent with the *in vitro *data; however, the in vitro data showed binding to AID^p^T is larger than AID^p^Y, which is not the case in the Biacore data.

X11 is able to bind APP, APLP1 and APLP2, and overexpression of X11α and X11β has been shown to decrease Aβ production in vitro and decreased amyloid deposition [80,109-112]. X11s also have roles in polarized trafficking in neurons and synaptic vesicle exocytosis [113,114]. The subcellular distribution of APP is changed when X11 is overexpressed in co-transfected non-neuronal cells, and its immunoreactivity was shown to be associated with AD plaques [115]. X11α has been seen to impair APP trafficking in secretory as well as endocytic pathways, which might lead to the prevention of Aβ secretion [116]. We found that X11α is phosphorylation independent, X11β shows higher binding to AID than its phosphorylated counterparts, and X11γ does not bind any of the peptides. This shows that most likely X11β may have a role in AD.

The general trend of the SH2 domains is that binding is seen when Tyr-682 is phosphorylated, and further increased when both the tyrosine and threonine are phosphorylated. The trend seen among the PTB domain proteins is either no difference of binding to AID and its homologues, regardless of phosphorylation, or a decrease in binding due to phosphorylation, especially by the tyrosine. Given the many roles of all the proteins mentioned, we felt there was a need to test the binding of a wide array of different proteins from kinases to scaffolds to signaling and regulatory proteins. This would allow us to see the many roles that APP could have, most of which are still unknown. Overall, our results indicate that phosphorylation of the cytoplasmic tail of APP on Tyr-682 and Thr-668 plays a role in the molecular composition of APP protein complexes. APP phosphorylation represents a "biochemical switch" that drastically changes the APP "interactome," creating docking sites for many of the domains discussed above (Figure 5). It also shows a second mechanism, alternative to APP processing by secretases, to regulate APP downstream signaling pathways. Given that APP is highly phosphorylated in AD cases, uncovering the mechanisms that regulate Tyr- 682 and Thr-668 phosphorylation and identifying the kinases and phosphatases that modify APP will lead to a better understanding of both biological and pathological brain processes. To this end, it is worth concluding that finding kinases and signaling pathways, such as the NGF-TrkA signaling pathway [117], that lead to APP phosphorylation is bound to have important biological and pathological consequences.

## Abbreviations used

APP: amyloid β protein precursor; Aβ: amyloid β peptides; AD: Alzheimer's disease; AID: APP intracellular domain; GST: glutathione S-transferase; SH2: Src homology domain 2; PTB: phosphotyrosine binding domain; WT: wild type.

## Competing interests

The authors declare that they have no competing interests.

## Authors' contributions

RT wrote the paper. DZ performed most of the experiments and cultures. LD conceived and designed the study, participated in the design of the experiments, participated in the analysis of the data, co-wrote the paper.
